# King Edward's Hospital Fund for London

**Published:** 1908-07-11

**Authors:** 


### KING EDWARD'S HOSPITAL FUND FOR LONDON.

King Edward's Hospital Fund has recently issued a circular to the hospitals of London reminding them of the resolution sanctioned by the General Council in 1903 to the effect—

That in future any new hospital or those reconstructing or extending to a considerable extent, within the area dealt with by the Fund, be requested before taking definite action to submit their proposals to the Fund.

The circular proceeds to set forth the full purport and intention of this resolution, and among other points states that the information submitted to the Fund should include the following: (1) Reasons and considerations which have led to the formulation of the proposals. (2) The nature of the proposals, with any outline sketche which may be necessary in order to make clear the main fatures of the site and buildings, but not detailed architects' plans unless specially asked for. (3) The amount of accommodation proposed to be provided, or other increase or modification of the work of the institution; compared so far as may be necessary with particulars of the present work. (4) The rental and tenure of the site or buildings. (5) The estimated cost of the proposals. (6) The resources from which it is anticipated the cost may be met. (7) The prospect of the provision for the increase (if any) in the cost of maintenance. (8) The approximate dates of the beginning and completion of the work.

Similar information is requested from the promoters of new hospitals who might contemplate an application to the Fund in the future. But it is pointed out that the Fund does not assist in the foundation of new hospitals or entertain applications for grants from hospitals that have not been in existence in a properly constituted form for a period of at least three years.

